# The Application of the Principle of Crown-Work to Metal Plates

**Published:** 1896-10

**Authors:** Joseph Head

**Affiliations:** Philadelphia


### THE APPLICATION OF THE PRINCIPLE OF CROWN-WORK TO METAL PLATES.

BY JOSEPH HEAD, M.D., D.D.S., PHILADELPHIA.

THE great difficuty in making gold dentures, when the teeth are ground to the plate, lies in the impossibility of grinding the teeth with sufficient accuracy to prevent an influx of food that will in time become foul. Although rubber attachments have to a certain extent lessened this objection to artificial dentures, still, when red rubber is used, its shrinkage from the porcelain and metal will invariably allow some small recess for leakage. And while pink rubber obviates the leakage to a great extent, the deterioration that invariably attends its prolonged presence in the mouth is a serious drawback.

My brother, Dr. L. F. Head, and I have been experimenting in this line for some years, and while our results are neither startlingly valuable nor original, still it would seem that we had solved the problem of making the porcelain lie absolutely against the gold of the plate. The ordinary process, as you know, is to strike up the plate, grind the teeth as closely to it as possible, back them, and solder the backing to the plate.

The variation from this would seem, now that it is done, comparatively simple. Instead of grinding the tooth to fit the articulation, it is ground accurately to the plate so as to be a trifle short. It is then backed up with a piece of thirty-two to thirty-four gauge annealed gold, which extends all the way between the plate and the porcelain to the cervical margin of the tooth. In burnishing this thin gold into the inequalities of the porcelain, the metal becomes springy and refuses to hug close to the tooth. When the backing has been trimmed to the size desired, it must be taken off and bent to an angle less than the angle made by the bottom and the side of the tooth, and when it is slipped in position again the gold and porcelain will lie snugly together everywhere.

Over this thin backing a thicker piece of plate is placed that covers the entire inside of the tooth, but does not extend between the plate and the porcelain, when the re-enforced backing can be secured by bending, splitting, or riveting the pins, as the mechanic may see fit.

In preparing the case for investing, the tooth should be so cemented to the plate that all cement can be easily removed with a fine instrument after the investment has become hard; and all around the cervical margin where the tooth backing touches the plate, a fine paste of borax should be placed to prevent the soft investment from marring the perfect filling in of the solder. Great care must also be taken that the plate and backing be well cleansed and borax used before the tooth is cemented in position. The investment of plaster and sand, or plaster and marble-dust, as may be desired, must be flowed over the tooth and around the plate with all the care one would exercise in investing a rubber denture; for if spaces are left where the solder can pinch the porcelain, chipping will infallibly occur.

When the investment is hard, pick away the cement, place the solder in position, and heat up from underneath until the solder starts of itself to flow, when, with the aid of gravity and the pointed flame, the solder will run down underneath the tooth and fill all the spaces solidly.

Where a tooth stands alone on the plate, the well-known principles of crown-work are to be followed in their severe simplicity; but when three or four teeth are to stand together side by side, the following precautions are imperative:

Each tooth must be so isolated by investment as to make it impossible for the solder to flow from one to the other, and spaces must be left between the teeth so that the contraction of the plate on cooling will not crush them against each other. Sometimes it has been found advisable to slip small pieces of mica in between the necks of the teeth, but it must not extend up high enough to jamb in between the porcelain. Where the teeth would naturally touch small pieces of paper should be placed, which, burning out under the flame, give a space that, while it is ample for safety, is quite imperceptible in the mouth.

When a single tooth is to be surrounded by solder, just as a rubber tooth is by rubber, all angles should be ground off the tooth where the solder could possibly cause the backing to pinch the porcelain; the backing should be cupped and invested around the tooth everywhere the solder is expected to flow; then, prior to splitting the pins, the backing is partially taken off and thin paper placed all around the edges between the metal and the porcelain. It is then slipped back into position and secured.

The tooth is then waxed to the case in the same way that one would wax a rubber case, care being taken not to flow the wax beyond the metal over the tooth.

This is then invested in the ordinary way, the wax boiled out, milk of borax wiped in with a brush, and the case is ready for filling in with solder, which is readily and safely accomplished if the heat is properly applied.

